# Genome-wide identification of basic helix-loop-helix (bHLH) genes in *Pinellia ternata* and their responses to abiotic stress and hormone treatments

**DOI:** 10.7717/peerj.21774

**Published:** 2026-09-30

**Authors:** Xiaorui Wang, Huirong Li, Zimo Li, Han He, Ziyu Xia, Yajun Liu, Xinghang Cai, Wenjing Huang, Xiaochun Sun

**Affiliations:** 1 Co-constructed Collaborative Innovation Center for Chinese Medicine Resources Industrialization by Shaanxi and Education Ministry, Shaanxi University of Chinese Medicine, Xianyang, China; 2 Yuxi Normal University, Yuxi, China

**Keywords:** *Pinellia ternata*, bHLH transcription factor, Genome-wide identification, Abiotic stress, Hormone response

## Abstract

**Background:**

Basic helix-loop-helix (bHLH) transcription factors constitute the second-largest transcription factor family in plants. They are widely distributed across plant genomes and play vital regulatory roles in plant growth and development, secondary metabolism, and responses to abiotic stress. Although the bHLH gene family has been widely identified and analyzed in many plant species, systematic genome-wide identification and functional analysis of bHLH genes in *Pinellia ternata* are still lacking.

**Results:**

In this study, a total of 140 *PtbHLH* genes were identified, of which 135 were unevenly distributed on 13 chromosomes. Phylogenetic analysis with *Arabidopsis thaliana* divided the *PtbHLH* genes into 24 subfamilies, with no members clustered in subfamily VI. Gene structure and conserved motif analysis showed that members of the same subfamily had highly similar structural characteristics. Collinearity analysis identified three paralogous pairs among the 140 *PtbHLH* genes. Cross-species collinearity analysis revealed eight syntenic pairs between *P. ternata* and *A. thaliana*, 76 between *P. ternata* and *Nicotiana tabacum*, and 50 between *P. ternata* and *Oryza sativa*. Cis-element prediction showed that all *PtbHLH* promoters contained hormone-responsive elements, stress-responsive elements and transcription factor binding sites. Gene Ontology (GO) and Kyoto Encyclopedia of Genes and Genomes (KEGG) enrichment indicated that *PtbHLH* genes were mainly involved in hormone signal transduction, environmental signal response and transcriptional regulation. Protein–protein interaction (PPI) analysis suggested that *PtbHLH68* was predicted as a putative interaction hub interacting with multiple family members. Tissue expression profiles showed that *PtbHLH* genes had diverse expression patterns, with most highly expressed in roots and tubers. Under high-temperature and drought stress, *PtbHLH9* was most sensitive to high temperature, and *PtbHLH85* was most sensitive to drought. Under methyl jasmonate (MeJA), abscisic acid (ABA) and salicylic acid (SA) treatments, *PtbHLH73* responded most strongly to MeJA, *PtbHLH7* to ABA, and *PtbHLH48* responded significantly to all three hormones. These four genes serve as core candidates for exploring *PtbHLH* functions in abiotic stress tolerance and hormone signal transduction.

**Conclusion:**

This study provides a solid foundation for understanding the functional mechanism of *PtbHLH* transcription factors in *Pinellia ternata*, and offers a valuable reference for the further screening of *PtbHLH* genes related to abiotic stress tolerance and hormone signaling pathways.

## Introduction

Basic helix-loop-helix (bHLH) transcription factors contain a conserved basic DNA-binding domain and a helix-loop-helix (HLH) dimerization domain. As the second-largest transcription factor superfamily in plants, bHLH proteins are widely distributed in plant genomes and play vital regulatory roles in plant growth and development, secondary metabolism, and abiotic stress responses (Ledent & Vervoort, 2001; Lei et al., 2024; Song et al., 2025). Heim et al. (2003) first classified 133 *Arabidopsis thaliana* bHLH proteins into 12 subfamilies based on structural features to facilitate functional prediction. In terms of growth and development, *SlMYB72* and *SlMYB75* antagonistically regulate tomato trichome formation and *SlCycB2* expression *via* the MYB-bHLH-WD40 complex (Qi et al., 2025); wheat *TaPGS1*, specifically expressed in grains 5–20 days post-anthesis, enhances grain weight/size and nutrient contents by activating *TaFl3* (Guo et al., 2022); maize *ZmBES1/BZR1-5* and *ZmBES1/BZR1-4* positively modulate grain development and yield (Feng et al., 2025). For secondary metabolism, *Betula platyphylla BpbHLH9* promotes triterpenoid accumulation by upregulating related biosynthetic genes (Yin et al., 2017); ABA/JA-induced *Artemisia annua AabHLH113* boosts artemisinin and dihydroartemisinic acid levels *via* targeting *DBR2* and *ALDH1* (Yuan et al., 2023); *Salvia miltiorrhiza SmbHLH148* activates phenolic acid and tanshinone biosynthesis through ABA/MeJA signaling (Xing et al., 2018). In abiotic stress responses, soybean *GmPIF4b* alleviates heat damage and preserves reproductive organ integrity under high temperatures (Arya, Singh & Bhalla, 2024); potato *StbHLH47* negatively regulates drought tolerance (Wang et al., 2025); SA enhances *Cinnamomum camphora* drought resistance by inducing stress-related genes and activating bHLH/MYB-mediated hormone signaling (Zhao et al., 2025). With maturing analysis technologies, genome-wide identification of transcription factor families has become a standardized bioinformatic workflow, and bHLH families have been identified in diverse plants, with member counts varying across species—164 in *Carya illinoinensis* (Zou et al., 2025), 122 in *Andrographis paniculata* (Xu et al., 2022), 83 in *Suaeda aralocaspica* (Wei, Cao & Lan, 2022), 12 in *Vitis vinifera* (Sun et al., 2025a), and 124 in *Chinese chestnut* (Li et al., 2025a)—laying a foundation for subsequent structural and functional studies of these genes.

*Pinellia ternata* (Thunb.) Breit., a perennial herb belonging to the *Pinellia* genus of the Araceae family, has a long history of medicinal use as its dried tuber, which is pungent, warm, and slightly toxic in nature, and acts on the lung, spleen, and stomach meridians with traditional efficacy in resolving dampness and phlegm, relieving vomiting, and dissipating abdominal masses (Liang et al., 2025). As a widely used bulk medicinal herb, the growth and development of *P. ternata* and the content of pharmacodynamic metabolites have attracted substantial attention. *P. ternata* mainly relies on asexual reproduction, which is closely associated with the formation and abscission of bulbils; high levels of jasmonic acid (JA), ABA, isopentenyladenosine (IPA), and SA have been detected in bulbils during their initial growth from petioles (Mou et al., 2024). During different growth and developmental stages of *P. ternata* corms, the contents of JA and ABA generally show an upward trend, which may enhance the corms’ adaptability to the environment during the transition from tender to mature tissues (Jia et al., 2025). *P. ternata* prefers shaded and humid growth conditions; sustained ambient temperatures above 35 °C or water-deficit (drought) stress will accelerate the senescence and death of its above-ground parts, leading to the “seedling lodging” phenomenon (Xue et al., 2019). High temperature and drought can cause damage to *P. ternata* petioles and leaves, resulting in abnormal physiological indices such as a significant increase in malondialdehyde (MDA) content (Liu et al., 2024), and studies have indicated that seedling lodging in *P. ternata* is essentially a form of programmed cell death. Alkaloids and nucleosides are the main bioactive components responsible for the medicinal effects of *P. ternata* (Chen et al., 2023); Duan et al. (2019) found that SA treatment of *in vitro* petiole culture systems for *P. ternata* microtuber induction significantly increased the contents of total alkaloids as well as guanosine, inosine, and ephedrine. In summary, high temperature, drought, and hormone treatments all exert notable impacts on the growth, development, and metabolism of *P. ternata*.

Despite widespread characterization of bHLH families across many plant taxa, no systematic research targeting the bHLH gene family has been reported for the medicinal herb *P. ternata* so far. Routine genome-wide identification pipelines alone cannot represent the innovation of this study; instead, the core value lies in the first comprehensive dissection of the bHLH repertoire specific to *P. ternata*. Using the publicly available *P. ternata* whole-genome assembly (Xue et al., 2024), we identified 140 *PtbHLH* family members and carried out multi-layered analyses covering physicochemical properties, gene and protein conserved architectures, phylogenetic relationships, intra- and interspecific collinearity, cis-acting regulatory elements, GO and KEGG functional annotations, and protein-protein interactions of these *PtbHLH* genes. Moreover, we analyzed the tissue-specific expression profiles of *PtbHLH* genes using public transcriptome datasets and selected representative genes to clarify their expression patterns under drought, high-temperature stress, and exogenous hormone treatments. This work provides a species-specific baseline for further functional exploration of bHLH regulators governing stress adaptation and medicinal metabolite biosynthesis in *P. ternata*.

## Materials and Methods

### Identification of *PtbHLH* gene family in *P. ternata*

Whole-genome sequence data of *P. ternata* with genome assembly Project ID PRJCA016404 and its corresponding gene annotation file were retrieved from the National Genomics Data Center (NGDC, https://ngdc.cncb.ac.cn/). *A. thaliana* bHLH protein reference sequences were downloaded from TAIR (https://www.arabidopsis.org). Two strategies were combined to capture candidate PtbHLH proteins: (1) BLASTp alignment against Arabidopsis bHLH sequences with E-value cutoff = 1e−5; (2) HMMER v3.0 screening using the bHLH hidden Markov model PF00010 from Pfam, with default gathering threshold as cutoff and a minimum conserved domain coverage of 70% retained. All candidate proteins were further verified for intact bHLH conserved domains *via* SMART (http://smart.embl.de) and NCBI CDD (https://www.ncbi.nlm.nih.gov/cdd/). Redundant protein sequences with 100% amino acid identity were removed using TBtools, and sequences lacking complete bHLH domains were eliminated to obtain the final set of 140 non-redundant PtbHLH proteins. TBtools was applied to predict molecular weight, theoretical isoelectric point and amino acid length of each PtbHLH protein (Chen et al., 2020), and subcellular localization was predicted *via* DeepLoc-2.1 (https://services.healthtech.dtu.dk/services/DeepLoc-2.1/).

### Phylogenetic analysis and chromosomal location

Conserved amino acid sequences of bHLH proteins from *P. ternata* and *A. thaliana* were subjected to alignment using the Muscle algorithm implemented in MEGA11. A Maximum Likelihood (ML) phylogenetic tree was constructed with 1,000 bootstrap replicates, which was subsequently visualized and refined *via* the online tool ChiPlot (https://www.chiplot.online). The *PtbHLH* family was classified with reference to the established *A. thaliana* bHLH classification system from prior studies. TBtools was used to extract chromosome length and positional information of *PtbHLH* members from the *P. ternata* genome sequence and GFF file (Chen et al., 2020).

### Motif, gene structure analysis of *PtbHLHs*

PtbHLH protein sequences were uploaded to the MEME server (https://meme-suite.org/meme/tools/meme) for conserved motif analysis, with the maximum number of motifs set to 10. TBtools was employed to visualize the genomic GFF3 file of *P. ternata* and the predicted conserved motifs.

### Cis-element analysis of *PtbHLHs*

Promoter sequences (2,000 bp upstream of *PtbHLH* genes) were retrieved with TBtools software, and cis-acting element prediction for these promoter sequences was performed using the PlantCARE database (http://bioinformatics.psb.ugent.be/webtools/plantcare/html/).

### Collinearity analysis between and within species

Genome data of three plant species (*A. thaliana*, *N. tabacum*, and *O. sativa*) were downloaded from Ensembl Plants (http://plants.ensembl.org/index.html). The One Step MCScanX-Super Fast module in TBtools was used to analyze the collinearity relationships within the *P. ternata* genome and between *P. ternata* and the three aforementioned species, with the analysis results visualized *via* TBtools (Chen et al., 2020).

### GO, KEGG, and interaction network analysis

GO and KEGG enrichment analyses of PtbHLH protein sequences were performed using TBtools, with the results visualized (Chen et al., 2020). Only the 140 PtbHLH proteins were set as the foreground gene set for enrichment, and the full *P. ternata* transcriptome was not adopted as the background reference. PPI network analysis was performed using the STRING database (https://cn.string-db.org/).

### Expression analysis of *PtbHLHs*

Published *P. ternata* RNA-seq data was downloaded from the NGDC website (https://ngdc.cncb.ac.cn/, accession number PRJCA016404) (Xue et al., 2024), covering different tissues (leaves, petioles, flowers, tubers, roots). Expression levels (transcripts per million, TPM) were calculated, and the results were visualized as heatmaps using the pheatmap package. One-month-old *P. ternata* plants were exogenously treated with 100 μM MeJA, 100 μM ABA, and 25 μM SA for 1 h, followed by transcriptome sequencing (NGDC accession number PRJCA059529) with three biological replicates per treatment. All RNA-seq data generated in this study are publicly available at NGDC under accession PRJCA059529. The statistical power of the hormone-treated transcriptome experiment was calculated using an RNA-seq power analysis tool. Parameters were set as follows: three biological replicates, average sequencing depth of 100 reads per gene, coefficient of variation (CV) of 0.4, fold change of two, and significance level α = 0.05, the calculated power was 0.48. TPM values under different hormone treatments were visualized using TBtools (Chen et al., 2020).

### Plant material and treatments

Uniform-sized *P. ternata* tubers were planted in the culture room of the Shaanxi Provincial and Ministerial Co-construction Center for Innovation in Chinese Medicinal Resources Industrialization, under controlled conditions of 25 °C and a 12 h/12 h light/dark cycle. For abiotic stress and hormone induction experiments, 1-month-old plants with three leaves were treated with 42 °C (high-temperature stress, short-term treatment without triggering seedling lodging and programmed cell death), 15% polyethylene glycol 6000 (PEG 6000) for drought simulation (seedlings only showed mild leaf curling within 24 h of treatment and could fully recover after rehydration, without severe lethal leaf necrosis), 100 μM MeJA, 100 μM ABA, and 25 μM SA, respectively. Leaves were harvested at 0, 1, 3, 6, 12, and 24 h post-treatment, promptly snap-frozen in liquid nitrogen, and stored at −80 °C for subsequent use. Each single biological replicate contained three pooled *P. ternata* plants, and the entire experiment was conducted with three independent biological replicates (nine individual plants in total per sampling time point); samples collected at 0 h served as untreated control groups. This experimental setup guaranteed reliable biological reproducibility.

### RNA isolation and quantitative reverse transcription polymerase chain reaction validation

Total RNA was isolated from various tissues of *P. ternata* using the TIANGEN (Beijing, China) Polysaccharide & Polyphenol Total RNA Extraction Kit (Catalog No. DP441). The isolated RNA was reverse-transcribed into cDNA in accordance with the manufacturer’s instructions for the TIANGEN (Beijing, China) Reverse Transcription Premix Kit (Catalog No. KR118). Specific primers for quantitative reverse transcription polymerase chain reaction (qRT-PCR) were designed using Primer 5 software based on the CDS sequences of 11 selected *PtbHLH* genes and synthesized by Sangon Biotech (Shanghai, China) (Table S1). With cDNA as the template and the 18S gene as the internal reference gene (Xue et al., 2019), qRT-PCR was performed using SuperReal PreMix Plus (SYBR Green, Catalog No. FP205-02) on the Analytik Jena qTOWER system. The PCR mixture consisted of 1 μL cDNA, 10 μL 2× SuperReal PreMix Plus, 0.6 μL of each primer, and 7.8 μL ddH_2_O. The qRT-PCR program included pre-denaturation at 95 °C for 15 s, followed by 40 cycles of denaturation at 95 °C for 10 s and annealing/extension at 60 °C for 32 s. After amplification, a melting curve procedure was performed: temperature was increased from 60 °C to 95 °C at a ramp rate of 0.5 °C/s, and fluorescence signals were continuously acquired during the temperature rise to verify specific amplification of target fragments. Each *PtbHLH* gene was detected based on three independent biological replicate samples. Relative gene expression levels were calculated using the 2^−ΔΔCt^ method (Livak & Schmittgen, 2001; Li et al., 2025b), and data were analyzed and visualized with GraphPad (Boston, MA, USA) Prism (v. 10.4.1).

## Results

### Identification of bHLH genes in *P. ternata* genome

A total of 140 bHLH gene sequences were identified through genome-wide analysis of *P. ternata* and named *PtbHLH1-PtbHLH140* sequentially (Table S2). The number of amino acids encoded by genes in this family ranged from 98 to 794 aa, with an average of 397 aa. The relative molecular weight varied from 11,367.59 to 88,279.59 Da, with a mean value of 43,034.07 Da. The theoretical isoelectric point (pI) spanned from 4.74 to 10.74, with an average of 6.60. Among these proteins, 96 had a pI below 7, accounting for 68.6% of the total and classifying them as acidic proteins. Only five members, *PtbHLH1/14/16/98/118*, had an instability index below 40, indicating that they are stable proteins. The aliphatic index ranged from 48.69 to 100.47, suggesting significant differences in thermal stability among family proteins. The grand average of hydropathicity (GRAVY) values were between −1.261 and 0.196, and all proteins were hydrophilic except *PtbHLH19*. Subcellular localization prediction showed that all PtbHLH proteins were localized in the nucleus, with *PtbHLH100* additionally localized in the cytoplasm.

### Phylogenetic analysis and chromosome location of the *PtbHLH* family

A ML phylogenetic tree was constructed using conserved amino acid sequences of 140 *P. ternata* bHLH proteins and 158 *A. thaliana* bHLH proteins (Fig. 1). Phylogenetic analysis clustered the total 298 bHLH protein sequences of two species into 25 subfamilies, among which 24 subfamilies contained *PtbHLH* genes and subfamily VI had no *P. ternata* bHLH members. Based on the phylogenetic tree, the 24 subfamilies containing *PtbHLH* genes were further grouped into 11 major clades. For qRT-PCR validation, one representative gene was selected from each of these 11 major clades, covering the full phylogenetic diversity of the family. Statistical analysis of the number of *bHLH* genes from *P. ternata* and *A. thaliana* in each subfamily was performed (Table S3). The results showed that no *PtbHLH* genes were detected in subfamily VI, implying potential gene loss or functional divergence during the evolution of *P. ternata* and subfamily XII contained the largest number of *PtbHLH* genes (21 in total). The number of *PtbHLH* genes in subfamilies IIIb, IIIc, IIId, IVd, Vb, VIIb, and XII was significantly higher than that of *AtbHLH* genes (ratio ≥1.50), implying that these subfamilies have undergone expansion during the evolution of *P. ternata* and may be associated with specific functional adaptations. In contrast, the gene number ratios of subfamilies Ib, IIIf, IX, VIIIa, and XI were ≤0.50, indicating a marked reduction in *PtbHLH* gene count in these subfamilies, which may result from gene loss or functional differentiation. For subfamilies Ia, IIIa, IIIe, and X, the gene number ratios were close to 1.00, suggesting that the gene numbers of these subfamilies have changed little between the two species and their functions may be more conserved. The 140 *PtbHLH* genes exhibited non-uniform distribution across 13 complete chromosomes and five unanchored scaffold fragments (Fig. 2). Among the 13 chromosomes, Chr09 harbored the most *PtbHLH* genes (20), followed by Chr02 (17), Chr10 (16), and Chr05 (15). In contrast, Chr12 (1) and Chr11 (3) contained the fewest genes. The gene numbers on the remaining chromosomes were intermediate: Chr01 (14), Chr03 (10), Chr04/Chr08 (6 each), Chr06 (12), Chr07 (11), and Chr13 (4).

**Figure 1:**
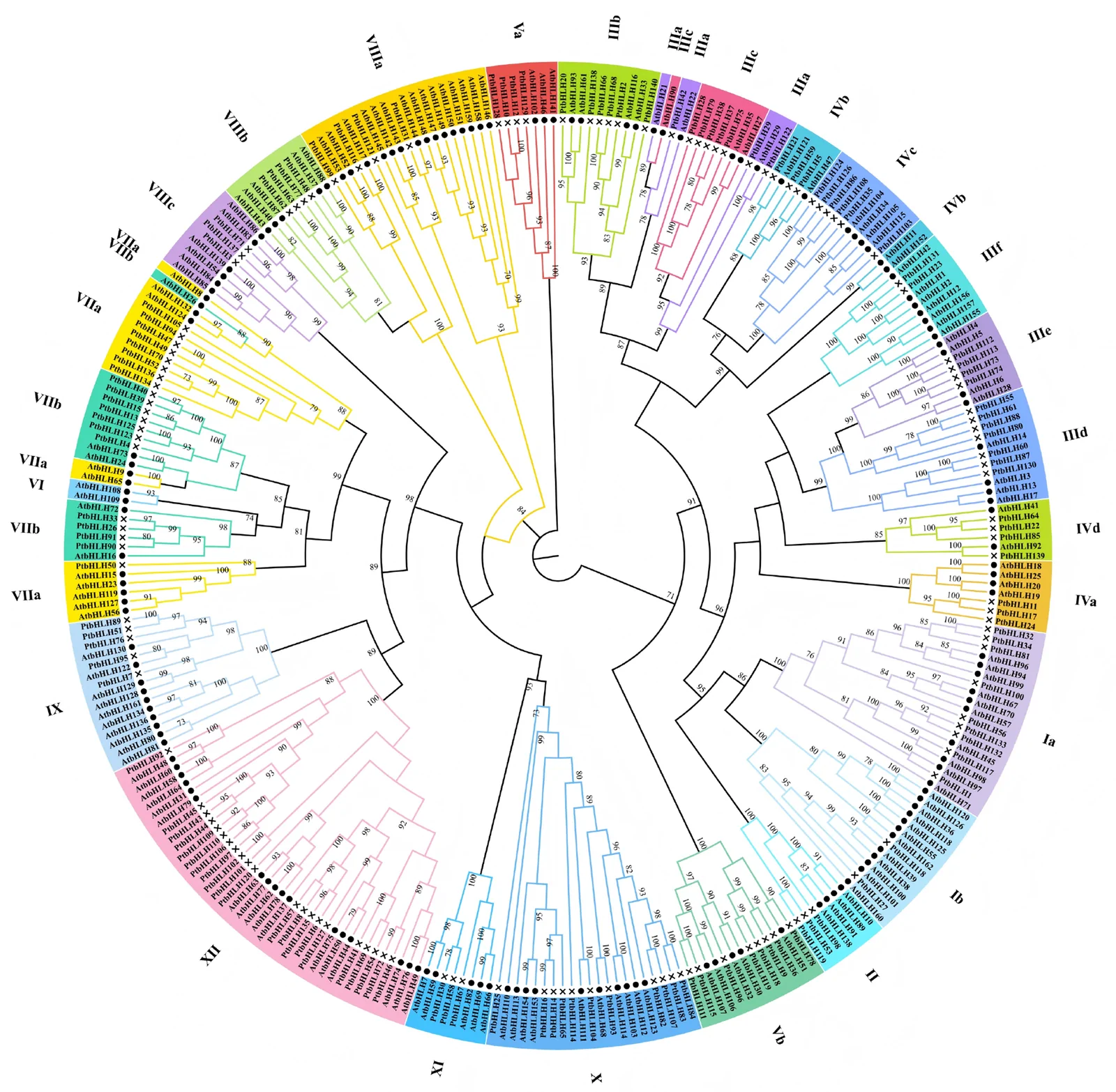
Phylogenetic tree of 140 PtbHLH and 158 AtbHLH proteins. Different background colors represent distinct subfamilies.

**Figure 2:**
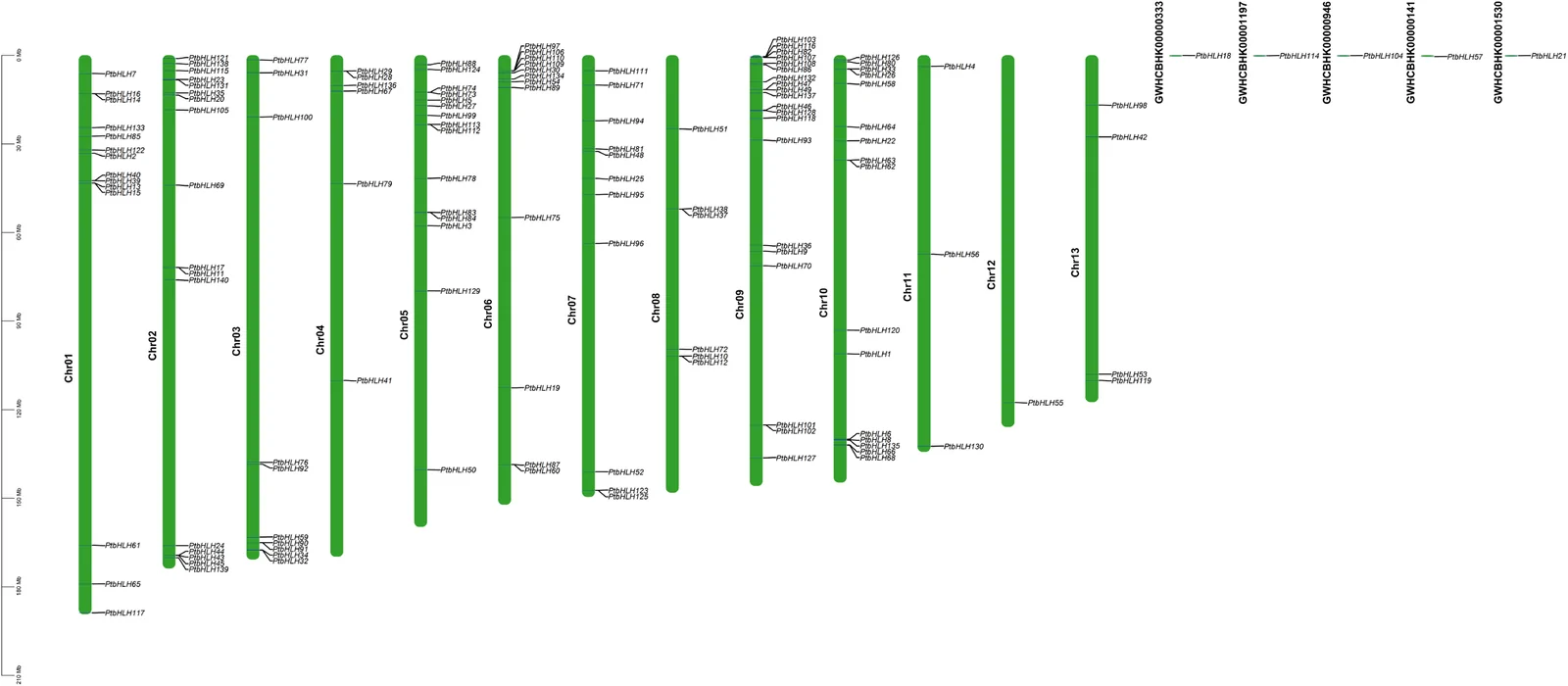
Chromosomal localization of *PtbHLH* genes.

### Analysis of gene structure, motif and conserved domains of *PtbHLHs*

To gain deeper insights into the sequence and structural characteristics of the *PtbHLH* gene family, a phylogenetic tree based on PtbHLH protein sequences was generated with MEGA11 (Fig. 3A). A motif distribution map was constructed through the MEME database (Fig. 3B), while detailed information regarding the composition of each motif is presented in Fig. S1. A total of 10 conserved motifs were identified, and the motif count in the 140 PtbHLH proteins varied from 1 to 10. Among them, motif 1 and motif 2 were the two most conserved motifs, widely distributed across *PtbHLH* family members and closely positioned in sequences (Fig. 3C). Conserved domain analysis *via* the SMART database revealed that all 140 PtbHLH proteins contained either the bHLH_AT or bHLH_SF domain (Fig. 3D). Gene structure analysis showed that some *PtbHLH* genes possessed multiple untranslated regions (UTRs), while others lacked these elements. The number of introns in *PtbHLH* genes varied dramatically, ranging from 0 to 9 (Fig. 3D). Generally, members within the same subfamily exhibited similar or identical gene structures, whereas significant differences were observed between different subfamilies.

**Figure 3:**
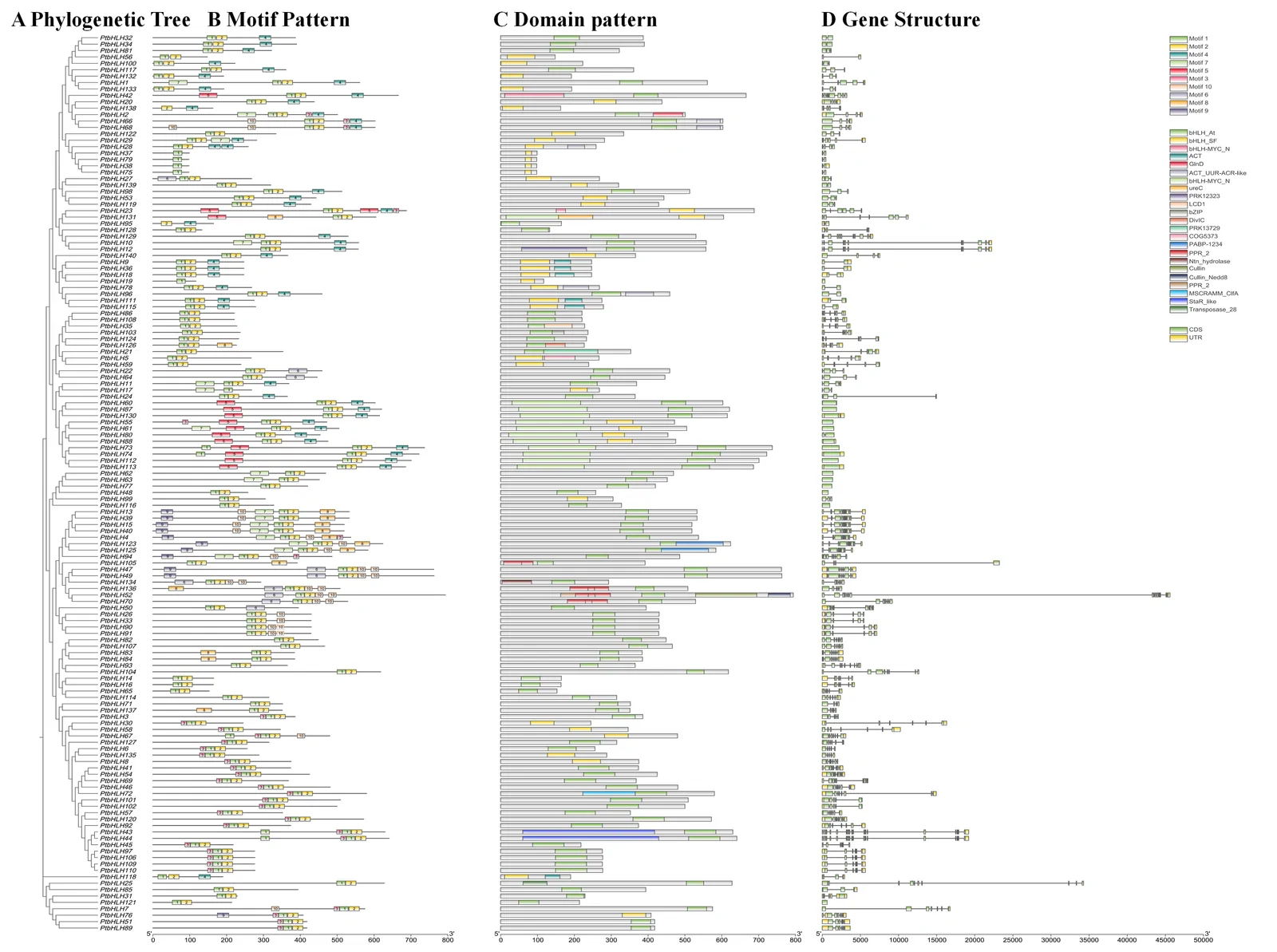
Phylogenetic and gene structure analysis of *PtbHLH* genes. (A) Phylogenetic tree of PtbHLH proteins. (B) Conserved motif analysis of PtbHLH proteins, with a total of 10 predicted motifs represented by different colored boxes. (C) Domain pattern analysis of PtbHLH proteins. (D) Gene structure analysis of *PtbHLH* genes.

### Cis-acting element analysis of the promoter region of *PtbHLHs*

Analysis of cis-acting elements in the promoter regions of 140 *PtbHLH* genes in *P. ternata* (Fig. 4) showed that these elements could be classified into three categories: growth and development-related, hormone-responsive, and abiotic stress and defense-responsive elements. Among growth and development-related elements, light-responsive elements (L) were detected in all *PtbHLH* genes and highly abundant in most of them (*e.g*., the L value of *PtbHLH25* and *PtbHLH129* reached 37), which are common motifs widely existing in plant promoters. Tissue- or process-specific elements such as cell cycle (Cyc), circadian rhythm (Circ), seed-specific (Sd), and root-specific (Rt) elements were only detected in a small number of genes (*PtbHLH11/14* contained Cyc elements; *PtbHLH14/16* contained Sd elements). Meanwhile, metabolism-related elements including MBSI (MYB-binding site involved in flavonoid biosynthesis) and Zein (zein metabolism-related) were also identified in partial members. For hormone-responsive elements, abscisic acid (ABRE), salicylic acid (SA), and methyl jasmonate (MeJA) responsive elements were widely distributed (*e.g*., the SA value of *PtbHLH25* reached 17; the MeJA value of *PtbHLH26* and *PtbHLH33* was 5), while auxin (Aux) and gibberellin (GA) responsive elements were relatively rare. In the category of abiotic stress and defense-responsive elements, salt stress-responsive elements (St) were highly enriched in some genes (the St value of *PtbHLH112* was 20; that of *PtbHLH1/22* was 16). Low-temperature-responsive (LT), anaerobic induction (Ana), and anoxic enhancement (Anx) elements were only present in a few genes as required. Additionally, MBS (MYB-binding site involved in drought inducibility) and Eli (elicitor activation-related) elements showed member-specific distribution patterns. Overall, the element composition of the *PtbHLH* gene family displayed characteristics of conserved core elements and differentiated specific elements, which only provides predictive clues for potential functional divergence rather than verifying that all *PtbHLH* members participate in hormone and stress signaling pathways. These cis-element features may be linked to the regulation of *P. ternata* growth and development, hormone signal transduction, and stress adaptation, offering candidate directions for subsequent gene function verification. Only qualitative classification and counting of cis-elements were performed here, without statistical functional annotation such as hypergeometric test against random promoter background sequences.

**Figure 4:**
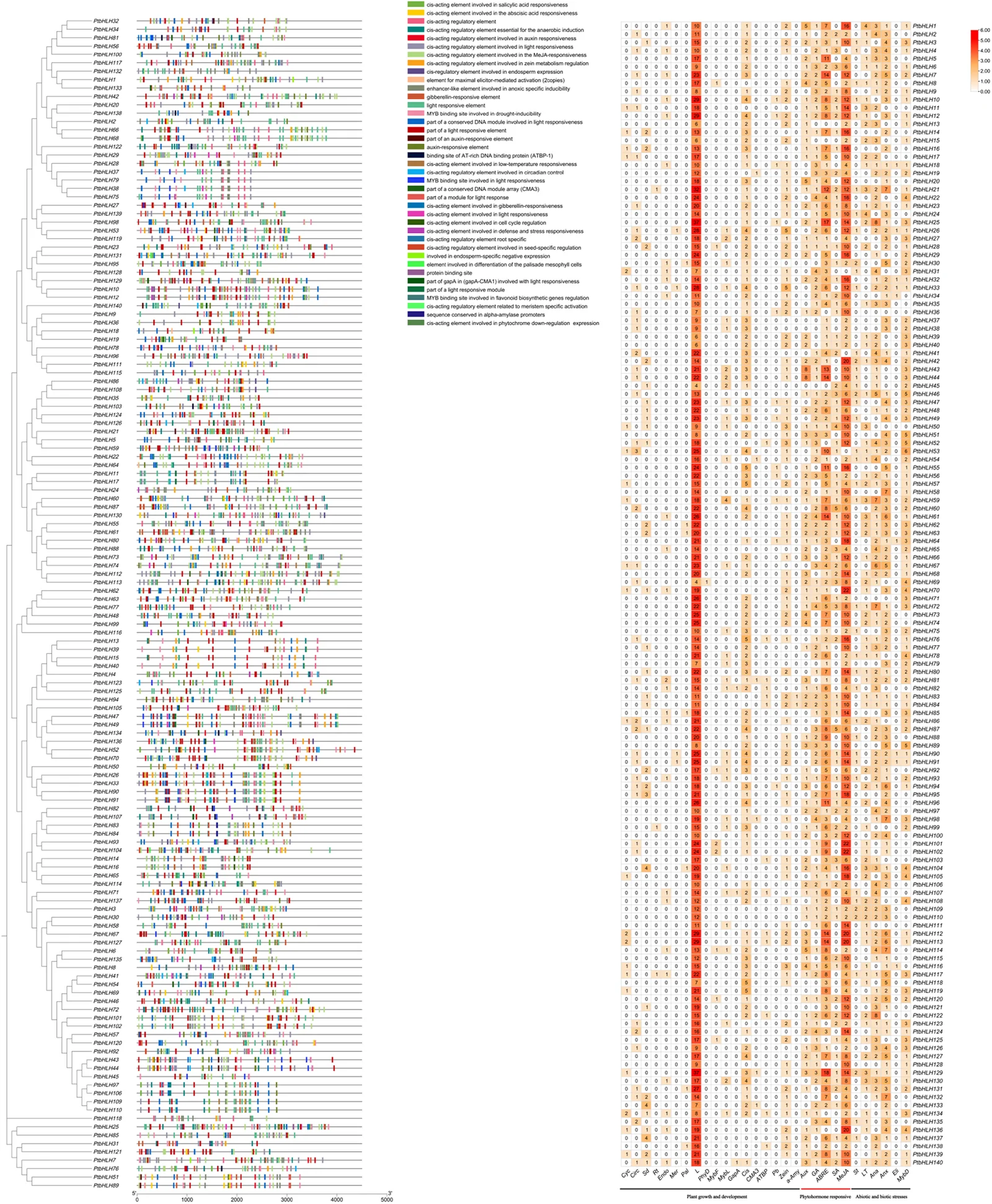
Analysis of cis-acting elements in the bHLH gene family of *P. ternata*. Numbers in grid cells represent the number of cis-acting elements.

### Gene duplication, and collinearity analysis of *PtbHLHs*

Three segmental duplication events were detected in the *PtbHLH* gene family (Fig. 5A, Table 1), involving the gene pairs *PtbHLH88/80*, *PtbHLH87/130*, and *PtbHLH64/2*. Notably, the Ks value of *PtbHLH87/PtbHLH130* reached 1.1736, implying that this pair derives from an ancient duplication with saturated synonymous substitutions. All these gene pairs exhibited Ka/Ks (non-synonymous substitution rate/synonymous substitution rate) <1, suggesting that the *PtbHLH* gene family has experienced purifying selection throughout its evolutionary process. Only one tandem duplicated pair (*PtbHLH28* and *PtbHLH29*) was screened out with criteria of interval <100 kb and protein identity >70%. To investigate the evolutionary relationships between bHLH genes of *P. ternata* and those from other plant species, interspecific collinearity analysis was conducted among *P. ternata, A. thaliana, N. tabacum*, and *O. sativa*. The results (Fig. 5B) showed that there were 8 collinear gene pairs between *P. ternata* and *A. thaliana*, 76 collinear gene pairs between *P. ternata* and *N. tabacum*, and 50 collinear gene pairs between *P. ternata* and *O. sativa*. These findings suggest that the bHLH gene family of *P. ternata* shares a closer homologous evolutionary relationship with that of *N. tabacum*.

**Figure 5:**
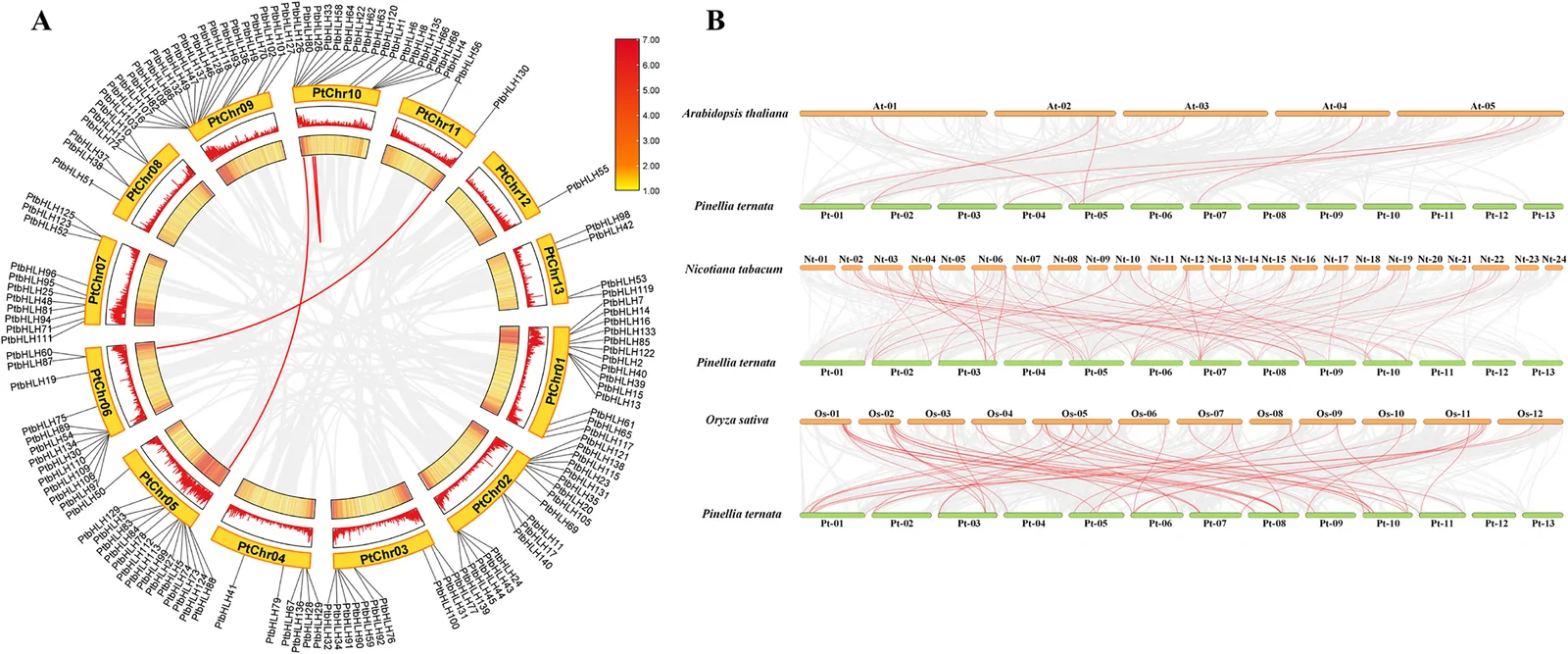
Collinearity analysis of *P. ternata bHLH* genes. (A) From outside to inside: chromosomes, gene density on chromosomes. (B) Collinearity between *A. thaliana*, *N. tabacum*, *O. sativa* and *P. ternata* (focusing on *bHLH* genes).

**Table 1:** Ka/Ks values of duplicated gene pairs in *P. ternata*.

GeneName1	GeneName2	Ka	Ks	Ka/Ks
*PtbHLH88*	*PtbHLH80*	0.2324	0.5438	0.4274
*PtbHLH87*	*PtbHLH130*	0.1840	1.1736	0.1568
*PtbHLH64*	*PtbHLH2*	0.0183	0.0395	0.4644

### GO, KEGG enrichment and protein-protein interaction analysis of *PtbHLHs*

GO enrichment was performed solely on the 140 *PtbHLH* family proteins, without the whole *P. ternata* transcriptome as background. GO functional annotation of the *PtbHLH* gene family in *P. ternata* (Fig. 6A) revealed the following key findings: In terms of molecular function, DNA-binding transcription factor activity, core promoter sequence-specific DNA binding, and DNA binding were the most significant terms. A total of 84 target genes were annotated to transcription factor activity-related terms, reflecting the inherent conserved characteristic of bHLH transcription factors in regulating gene expression. For cellular component, the nucleus was the major enriched localization, with 68 genes localized therein—consistent with the subcellular localization requirement for transcriptional regulation. For biological process, the genes were significantly enriched in nucleic acid metabolism, RNA metabolism, and pathways associated with transcriptional regulation. KEGG enrichment results (Fig. 6B) indicated that the genes were mainly concentrated in pathways such as signal transduction, plant hormone signal transduction, transcription factors, plant MAPK signaling pathway, and plant circadian rhythm. This further suggests that *PtbHLH* genes may modulate the growth and development, stress adaptation, and secondary metabolism of *P. ternata via* hormone signal transduction, environmental signal response, and transcription regulation, offering potential clues for follow-up functional research.

**Figure 6:**
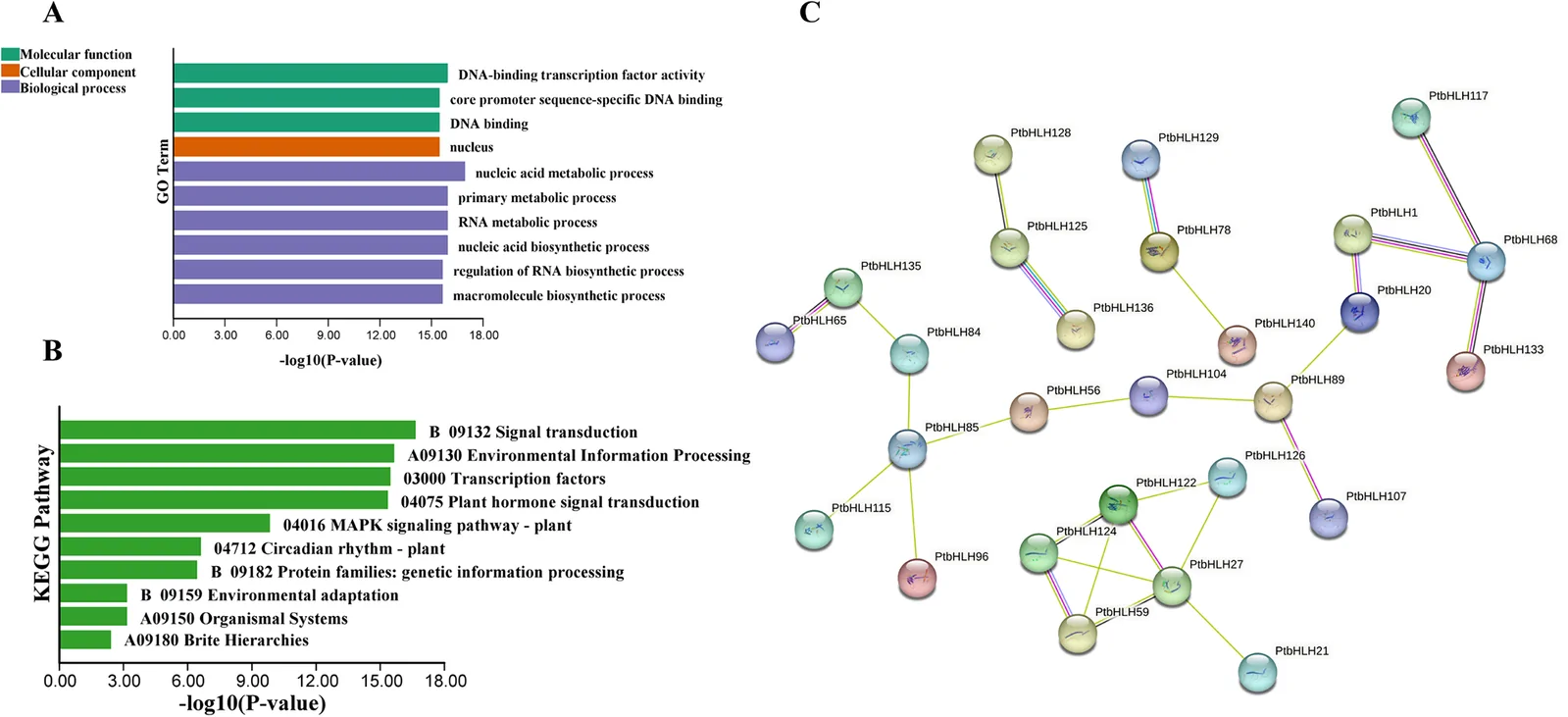
GO, KEGG enrichment and regulatory network analysis of *PtbHLH* genes. (A) GO functional annotation of *PtbHLH* genes. (B) KEGG functional annotation of *PtbHLH* genes. (C) PtbHLH protein-protein interaction analysis based on *A. thaliana* homologous genes. Among the lines in (C): purple lines represent experimentally determined interactions; blue lines represent interactions from curated databases; yellow lines represent text-mining evidence; black lines represent co-expression relationships; light purple lines represent interactions inferred from protein homology.

Screening and interaction prediction of 140 *bHLH* gene sequences in *P. ternata* were conducted using two criteria: sequence identity with *A. thaliana* bHLH proteins >40% (homology standard) andinteraction score >0.7 (credibility threshold). Finally, 27 *PtbHLH* members with mutual regulatory relationships were obtained (Fig. 6C). These members formed a modular protein interaction network: for example, *PtbHLH17/1/20/68/133/122/124/59/27*, and *PtbHLH135/65/84/85* respectively formed multiple dense interaction clusters. Some members (*e.g*., *PtbHLH68*) served as “core nodes” interacting with multiple members, which are predicted to be key targets of the regulatory mechanism of this gene family. It should be noted that the 40% identity cutoff may lead to false-positive interactions, and all predicted protein interactions need further experimental verification such as Y2H or Co-IP in subsequent work.

### Expression pattern of *PtbHLHs* in different tissues and hormone treatments of *P. ternata*

Members of the *PtbHLH* family exhibited divergent tissue-preferential expression across five tissues (leaf, petiole, flower, tuber, root) (Fig. 7A), and their expression preferences were closely linked to tissue identity. *PtbHLH4*, *PtbHLH31* and *PtbHLH104* displayed abundant transcript accumulation in leaves; *PtbHLH26*, *PtbHLH57* and *PtbHLH120* were highly transcribed in petioles. *PtbHLH35* showed the highest expression level in flowers, accompanied by moderate expression of *PtbHLH21*, *PtbHLH77* and *PtbHLH50*, while *PtbHLH100* was exclusively detected in flowers as a flower-preferential gene. *PtbHLH2*, *PtbHLH66* and *PtbHLH125* represented the dominant highly expressed genes in tubers, and *PtbHLH18*, *PtbHLH28* and *PtbHLH139* showed relatively high transcript abundance in roots. A subset of genes displayed tissue-restricted transcriptional absence: *PtbHLH1* was undetectable in flowers and roots, with only weak expression in leaves, petioles and tubers; *PtbHLH6* had extremely low transcript levels solely in roots and was absent from all other tissues; *PtbHLH71* maintained negligible or undetectable expression across all examined tissues. These distinct expression profiles reflect tissue-based functional differentiation within the *PtbHLH* family. Genes with TPM < 1 were regarded as transcriptionally undetectable in corresponding tissues.

**Figure 7:**
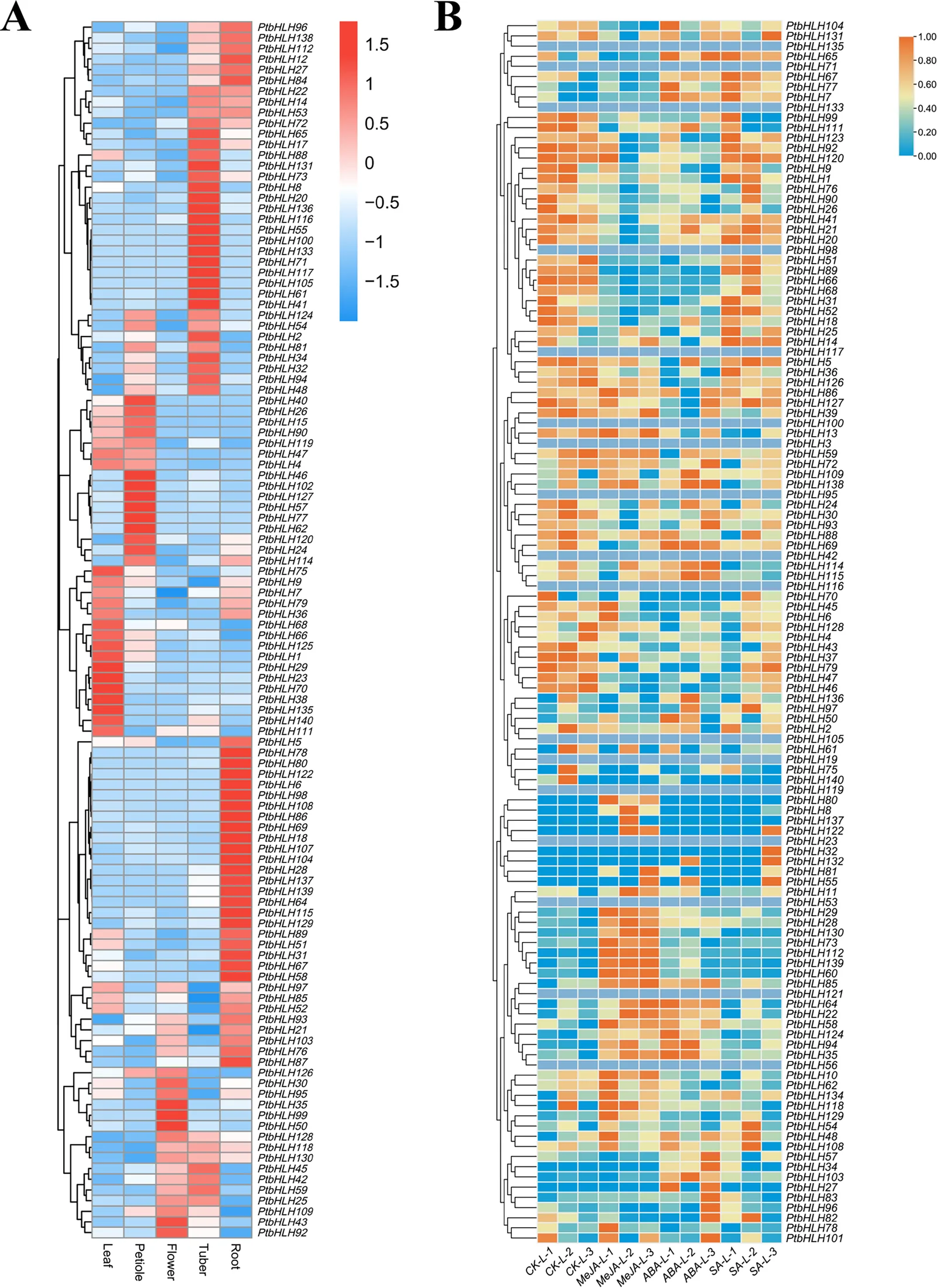
Expression analysis of *PtbHLH* genes. (A) Tissue-specific expression patterns of *PtbHLH* genes in five tissues (leaf, petiole, flower, tuber, root) of *P. ternata*. (B) Expression changes of *PtbHLH* genes in *P. ternata* leaves under different hormone treatments (100 μM MeJA, 100 μM ABA, 25 μM SA) for 1 h.

Transcriptome sequencing was conducted on 1-month-old *P. ternata* leaves harvested 1 h after exogenous treatment with 100 μM MeJA, 100 μM ABA and 25 μM SA (Fig. 7B). This dataset only captured short-term early transcriptional shifts of the 140 *PtbHLH* family members upon hormone treatment; full-transcriptome differential expression statistical testing was not performed, and the heatmap was generated purely based on extracted TPM values of bHLH genes. Under MeJA treatment, *PtbHLH73*, *PtbHLH112*, *PtbHLH139*, *PtbHLH60* and *PtbHLH85* presented clearly elevated TPM values, whereas *PtbHLH51*, *PtbHLH89*, *PtbHLH66*, *PtbHLH68* and *PtbHLH31* showed obvious reductions in transcript abundance. Following ABA treatment, *PtbHLH7*, *PtbHLH85*, *PtbHLH64*, *PtbHLH22* and *PtbHLH103* displayed increased TPM levels, while *PtbHLH89*, *PtbHLH66*, *PtbHLH68*, *PtbHLH126* and *PtbHLH46* exhibited reduced transcript accumulation. After SA treatment, elevated TPM signals were observed for *PtbHLH67*, *PtbHLH77* and *PtbHLH7*, and decreased transcript levels were detected for *PtbHLH115*, *PtbHLH24* and *PtbHLH13*. Overall, the magnitude of TPM variation triggered by 1 h SA exposure was weaker than the transcriptional fluctuations induced by MeJA and ABA. Several genes including *PtbHLH64*, *PtbHLH22*, *PtbHLH85*, *PtbHLH89*, *PtbHLH66* and *PtbHLH68* shared similar TPM alteration trends under short-term MeJA and ABA treatments. To characterize dynamic expression responses over extended timeframes, 11 representative genes from distinct evolutionary clades were selected for time-series qRT-PCR detection covering 0–24 h. The qRT-PCR results revealed delayed transcriptional responses at 6–24 h for multiple candidate genes; nevertheless, these quantitative expression patterns only apply to the 11 selected representatives and cannot be extrapolated to all members of the *PtbHLH* gene family.

### Response of *PtbHLHs* to abiotic stress and hormone treatment

To investigate the response characteristics of *PtbHLH* genes to abiotic stresses and plant hormones, this study used 1-month-old *P. ternata* as materials, and conducted treatments including high temperature (42 °C), drought (15% PEG6000), 100 μM MeJA, 100 μM ABA, and 25 μM SA, to analyze the expression dynamics of 11 *PtbHLH* genes (one selected from each of the 11 major evolutionary clades) in leaves (Fig. 8). Under 42 °C high-temperature stress: *PtbHLH9/27/48/65/67/127* showed no significant upregulation at 1 and 3 h of treatment, with their expression peaks delayed until 12 or 24 h; *PtbHLH53* was significantly induced at 3 h; *PtbHLH73/76/90* were significantly downregulated at 1 h, then gradually increased with prolonged treatment duration, reaching peaks at 12 or 24 h; *PtbHLH85* had low expression at 1 and 3 h, significantly increased from 6 h onwards, and maintained high-level expression after 12 h. Notably, *PtbHLH9* showed extremely significant response to 42 °C high temperature, with the expression increase amplitude at the peak stage significantly higher than that of some other members in the family, implying it may be a candidate gene associated with heat stress response. Under 15% PEG6000-simulated drought treatment: most genes generally showed expression inhibition at 1 and 3 h, their relative expression levels significantly increased at 6 and 12 h, and significantly decreased at 24 h; *PtbHLH53* had the basal expression at 0 h and maintained low levels after treatment; *PtbHLH76* had a relatively gentle response pattern with no significant fluctuations in expression; most genes including *PtbHLH9/27/48* reached expression peaks at 12 h, suggesting that 12 h may be a key time point for drought response.

**Figure 8:**
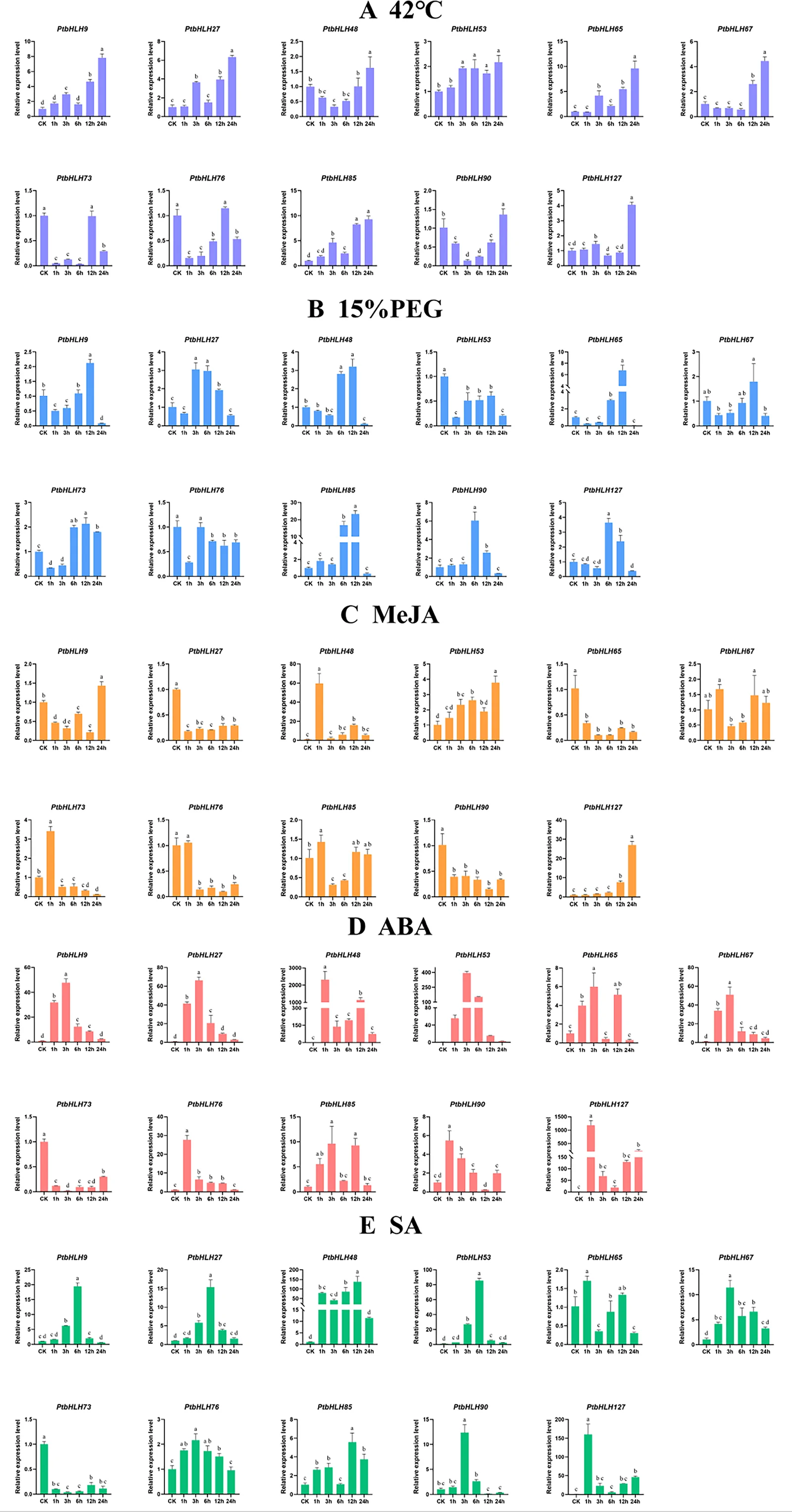
Expression patterns of 11 *PtbHLH* genes in *P. ternata* leaves under abiotic stress and hormone treatments. The treatment methods are as follows: (A) High temperature (42 °C); (B) drought (15% PEG6000-simulated drought); (C) 100 μM MeJA; (D) 100 μM ABA; (E) 25 μM SA. Error bars indicate the ± SD of three independent experiments. One-way ANOVA was performed after Brown–Forsythe/Bartlett variance homogeneity tests; *post-hoc* comparisons used Tukey’s HSD test. Different lowercase letters (*P* < 0.05) denote significant differences.

Analysis of the responses to plant hormones MeJA, ABA, and SA showed that these three hormones may participate in regulating plant growth and development. Under MeJA treatment: *PtbHLH9/53/127* reached expression peaks at 24 h; *PtbHLH27/65/76* had the highest expression at 0 h untreated control, followed by a downward trend. It is worth noting that *PtbHLH48* and *PtbHLH73* were significantly induced at 1 h of MeJA treatment, and their expression levels continued to decrease significantly thereafter—among them, the expression level of *PtbHLH48* increased to approximately 60-fold that at 0 h, which may suggest that this gene participates in MeJA signal transduction. Under ABA treatment: except for *PtbHLH73*, all other genes were rapidly upregulated to peaks at 1 or 3 h, then significantly downregulated with prolonged time; *PtbHLH73* had high expression at 0 h, decreased sharply after 1 h and maintained low levels, while *PtbHLH48* and *PtbHLH127* were most significantly induced by ABA, with their expression levels reaching thousands of times that at 0 h. Under SA treatment: most genes including *PtbHLH9/27/53* showed a trend of first gradually increasing, reaching peaks at 3 or 6 h, then gradually decreasing—among them, *PtbHLH48* and *PtbHLH127* had significant induction effects, with their expression levels reaching 150-fold that at 0 h at a later time point, which may reflect the functional differentiation of this gene family in hormone signal response.

## Discussion

Genome-wide identification of transcription factor families has become a routine bioinformatic pipeline in plant genomics, and the novelty of the present study does not lie in this standard identification workflow itself. Instead, its core value lies in the first systematic characterization of the bHLH gene family in the medicinal herb *P. ternata*, a species lacking prior relevant reports. The bHLH transcription factor family is among the largest transcription factor families in plants. It plays crucial roles in regulating plant growth and development, as well as in responses to abiotic stresses (Heim et al., 2003). To date, *bHLH* gene families have been reported in plants such as *A. thaliana, O. sativa* (Li et al., 2006), *N. tabacum* (Bai et al., 2021), *Panax ginseng* (Zhu et al., 2020), *Scutellaria baicalensis* (Sun et al., 2025b), and *Perilla frutescens* (Chen et al., 2024); however, no such report has been published for the medicinal plant *P. ternata*. This study identified 140 members of the *PtbHLH* family in *P. ternata*. The number of bHLH family members varies among different plants, which may be related to genome duplication events, evolutionary difference, or genome size variations among plant species (Qiao et al., 2019)—for example, there are 41 bHLH family members in *Carthamus tinctorius* (Yingqi et al., 2019), while there are 448 bHLH family members in *Phyllostachys edulis* (Cheng et al., 2018). Among the *PtbHLH* genes in *P. ternata*, 135 are unevenly distributed on 13 chromosomes, and five genes are located on unanchored scaffold fragments. Genome sequencing results of *P. ternata* show that the total length of the 13 chromosomes accounts for 95.46% of the total genome sequence (Xue et al., 2024), which may be caused by incomplete genome assembly.

At present, the biological functions of most *PtbHLH* members remain unclear. Analysis of their gene structure, motifs, and conserved domains shows that members of the same subfamily have similar structures and conserved motifs, indicating that members of the same subfamily may have certain functional similarities. Different *P. ternata bHLH* genes contain different numbers of exons and introns; however, in subfamilies IIIe and VIIIb, none of the *P. ternata bHLH* genes have introns, which supports the certain accuracy of subfamily classification. Ten conserved motifs were identified in the 140 *P. ternata* bHLH protein sequences. Among them, conserved Motif 2 is present in all *P. ternata* bHLH protein sequences, suggesting that it plays an irreplaceable role in the function of *P. ternata bHLH* genes. Through phylogenetic analysis of *A. thaliana* bHLH proteins, the *bHLH* family was classified into 24 subfamilies. No *P. ternata bHLH* genes were found in subfamily VI, indicating that partial differentiation has occurred between the *bHLH* gene families of *P. ternata* and *A. thaliana* during long-term evolution. This phenomenon has also been observed in plants such as *Atractylodes lancea* (Zhang et al., 2025), *Lilium bakerianum* var. *rubrum* (Gu et al., 2025), and *Zanthoxylum armatum* (Yi et al., 2025).

Cis-acting elements in the promoter region play an important role in regulating gene function (Fan et al., 2025). The cis-acting elements in the promoter region of *P. ternata bHLH* genes are mainly classified into three categories: growth and development-related, hormone-responsive, and abiotic stress-responsive elements. Every *PtbHLH* gene contains light-responsive elements, indicating that *PtbHLH* genes are regulated by light signals and are of great significance for coordinating plant growth and development. Most *PtbHLH* genes contain MeJA, SA, and ABA responsive elements. MeJA has a significant inductive effect on the accumulation of various plant secondary metabolites and can also act synergistically with other hormones such as ABA. In *Artemisia annua* (sweet wormwood), *AaMYC2* and *AabHLH113* induced by JA and ABA signals, respectively, activate *CYP71AV1*, double bond reductase (*DBR2*), and *AaALDH1*, which positively regulate the biosynthesis of artemisinin (Yuan et al., 2023). In terms of abiotic stress, studies have found that the enhancement of plant drought resistance induced by bHLH transcription factors is generally related to the signal transduction of ABA and JA pathways and the scavenging of reactive oxygen species (ROS) (Li et al., 2007). In *Triticum aestivum*, over expression of *TabHLH1* can alter growth characteristics such as stomatal movement and leaf water loss rate under drought stress, and improve drought adaptability through the ABA pathway. Based on this, it is speculated that under hormone induction (such as JA and ABA) and drought stress, *PtbHLH* genes homologous to *AaMYC2* and *TabHLH1* may have similar functions.

GO functional annotation showed that *PtbHLH* genes are mainly localized in the nucleus, have DNA-binding ability, and are involved in primary metabolism and macromolecular biosynthesis processes. KEGG functional annotation indicated that *PtbHLH* genes are mainly involved in environmental information processing and plant hormone signal transduction processes. These enrichment results confirm that as transcription factors, *P. ternata PtbHLH* genes exert their functions by regulating other genes when affected by the external environment or induced by hormones, thereby maintaining the normal growth and development of plants. Analysis of the interaction relationship of PtbHLH proteins showed that *PtbHLH68* was predicted as a putative interaction hub and interacts with multiple members. It is predicted that this protein may be a key target of the regulatory mechanism of the *PtbHLH* family.

The tissue-specific expression patterns of *PtbHLH* family genes in *P. ternata* reflect the functional differentiation of this family formed during evolution. The five tissue types (leaf, petiole, flower, tuber, root) all have core highly expressed members, and their expression preferences are closely associated with the physiological functions of each tissue: highly expressed genes in leaves may be involved in the regulation of photosynthesis; *PtbHLH100* (flower-specific) and highly expressed *PtbHLH35/21* in flowers are likely to play key roles in reproductive development; while core genes in tubers and roots may mediate processes such as nutrient storage, absorption, and transport. Transcriptome sequencing was performed on *P. ternata* leaves after 1 h of MeJA, ABA, and SA treatments. In the MeJA treatment group, the expression levels of genes such as *PtbHLH73/112/139/60* were significantly increased, among which *PtbHLH73/112* are *MYC2* homologous genes. *MYC2* genes responsive to MeJA have been reported in multiple plant species (Li et al., 2024). In *Taxus chinensis*, the JA-responsive transcription factor *TcMYC2a* directly regulates the expression of ERF transcription factors such as *TcERF15* to control *TcTASY* expression, thereby promoting paclitaxel biosynthesis (Zhang et al., 2018). In *Aquilaria sinensis*, *AsMYC2* regulates the expression of *ASS1* through the JA signaling pathway and participates in the regulation of sesquiterpene biosynthesis (Xu et al., 2017). In *Catharanthus roseus*, overexpression of *CrMYC2* reduces alkaloid accumulation in roots (Sui et al., 2018). Growing evidence indicates that genes related to the biosynthesis of active components also show tissue-specific expression patterns, *PtMYC2* (*PtbHLH73/112*) is highly expressed in *P. ternata* tubers which are rich in components such as alkaloids and, nucleosides. Based on this, it is speculated that *PtMYC2* (*PtbHLH73/112*) in *P. ternata* may respond to JA signals and regulate alkaloid synthesis in *P. ternata*. In the ABA treatment group, the expression levels of genes such as *PtbHLH52/66/68/126* changed significantly, among which *PtbHLH52* is a *CUL4* homologous gene. In *A. thaliana*, *DWA1* and *DWA2* are two DWD protein components of *CUL4*-based E3 ligases, and they jointly act as negative regulators in ABA signal transduction (Lee et al., 2010); similarly, *PtbHLH52* in *P. ternata* is also negatively regulated by ABA signals. In the SA treatment group, the expression of *PtbHLH* genes showed no obvious changes. This may be due to the insensitivity of *P. ternata* leaves to SA induction, resulting in insignificant changes at 1 h; significant differences may occur at later time points. Notably, the statistical power of the hormone-treated RNA-seq experiment was 0.48, which is below the commonly accepted threshold of 0.8. This indicates that the current number of biological replicates limits the ability to reliably detect genes with moderate expression changes. Therefore, we interpreted all non-significant expression results with caution. Increasing the number of biological replicates will be adopted in future related studies to improve the statistical power and detection capability.

High temperature, drought stress, and hormone induction have significant impacts on the growth of *P. ternata*. To further explore the role of *PtbHLHs* in the above environments, this study analyzed the expression profiles of 11 *PtbHLHs* under high temperature, drought stress, and hormone induction. Under high-temperature stress: Silencing *LibHLH87* in *Lilium* reduced its basal heat tolerance, while *LibHLH871* could activate *LlEIN3* and *LlHSFA2* to improve heat tolerance in *Lilium* (Wu et al., 2024). In *P. ternata*, *PtbHLH9* showed a characteristic of significantly increased expression in response to high temperature, indicating that it may act as a core node in the heat tolerance regulatory network of *P. ternata* by activating the transcription of downstream heat shock proteins (HSPs) or genes related to stress-resistant metabolic pathways; in contrast, some genes (*PtbHLH67/127*) showed delayed responses, which may be associated with cell repair under long-term high-temperature stress. Under drought stress: Most genes exhibited an expression pattern of “decrease first, then increase, then decrease again”, which was basically consistent with the activation sequence of the plant osmotic adjustment mechanism. However, *PtbHLH76* showed a relatively gentle response pattern, suggesting that it may have functional redundancy or a specific regulatory role in drought stress. At the hormone response level: The expression trends of *PtbHLH* genes after 1 h of MeJA, ABA, and SA treatments were basically consistent with the transcriptome data. Notably, *PtbHLH48* was an exception—its expression showed no significant changes under the three hormone inductions in the transcriptome results, but was significantly highly expressed in qRT-PCR experiments. This inconsistency is mainly caused by independent biological samples and different detection sensitivity of the two techniques. As a homologous gene of *HEC2*, *HEC1* has been found in *A. thaliana* to act as a local regulator of auxin and cell division protein responses to control female cell development in *Arabidopsis* (Schuster, Gaillochet & Lohmann, 2015); however, its association with other hormones has not been studied yet, which is worthy of in-depth exploration in subsequent research.

In summary, we systematically characterized the bHLH gene family specific to the medicinal plant *P. ternata via* multi-dimensional bioinformatic analysis and expression profiling, and screened key candidate genes responding to high temperature, drought, MeJA, ABA and SA. The unique evolutionary features, cis-element distribution and tissue/stress expression patterns of *PtbHLHs* identified here fill the research gap of bHLH transcription factors in this medicinal species. These results lay a foundation for uncovering the species-specific molecular regulatory mechanisms of *PtbHLHs* in abiotic stress tolerance and hormone signal transduction, and provide potential genetic targets for stress resistance improvement and molecular breeding of *P. ternata*. For future research, molecular biology techniques such as gene overexpression and gene knockout will be used to verify the potential biological functions of expression-supported candidate genes including *PtbHLH9*, *PtbHLH85*, *PtbHLH73*, *PtbHLH7* and *PtbHLH48* in *P. ternata*, as no direct functional validation was performed in the current study. Further experiments covering cold and salt stress treatments will also be supplemented to explore their expression response patterns. In addition, metabolite quantification of alkaloids and nucleosides under different treatments will be conducted in follow-up work to establish the correlation between the expression of *PtbHLHs* and the accumulation of medicinal active ingredients, which is a limitation of the present study. Another limitation is that only 18S rRNA served as the qRT-PCR reference gene, and no no-RT controls were included. Further assays with multiple reference genes and no-RT controls will validate these expression results.

## Conclusions

The bHLH family has been widely characterized across plants. Here, 140 *PtbHLH* genes were identified from the *P. ternata* genome and divided into 24 subfamilies *via* phylogenetic analysis with *A. thaliana*. We systematically analyzed their chromosome distribution, gene structure, conserved domains, promoter cis-elements, collinearity, duplication events, GO/KEGG enrichment and protein-protein interactions. Tissue-specific expression patterns suggest diverse developmental functions of *PtbHLH* members in *P. ternata*. qRT-PCR under heat and drought stresses identifies *PtbHLH9* as a heat-responsive gene and *PtbHLH85* as a drought-responsive gene. Hormone treatment assays further identify key responsive genes: *PtbHLH73* for MeJA, *PtbHLH7* for ABA, and *PtbHLH48* for MeJA, ABA and SA. This work characterizes the structural features and expression patterns of the *PtbHLH* family, provides clues for studying its regulatory mechanisms in abiotic stress and hormone responses, and offers insights for molecular breeding of high-quality *P. ternata*.

## Supplemental Information

10.7717/peerj.21774/supp-1 Supplemental Information 1 Conserved motif logos of *PtbHLH* family in *P. ternata*.

10.7717/peerj.21774/supp-2 Supplemental Information 2 qRT-PCR primers for *PtbHLH* genes in *Pinellia ternata*.

10.7717/peerj.21774/supp-3 Supplemental Information 3 Physicochemical properties of PtbHLH proteins in *P. ternata*.

10.7717/peerj.21774/supp-4 Supplemental Information 4 Subfamily distribution of bHLH genes in *P. ternata* and *A. thaliana*.

10.7717/peerj.21774/supp-5 Supplemental Information 5 MIQE Checklist.

10.7717/peerj.21774/supp-6 Supplemental Information 6 qPCR raw data MeJA *PtbHLH*.

10.7717/peerj.21774/supp-7 Supplemental Information 7 qPCR raw data ABA *PtbHLH*.

10.7717/peerj.21774/supp-8 Supplemental Information 8 qPCR raw data SA *PtbHLH*.

10.7717/peerj.21774/supp-9 Supplemental Information 9 qPCR raw data Drought *PtbHLH*.

10.7717/peerj.21774/supp-10 Supplemental Information 10 qPCR raw data Heat *PtbHLH*.

10.7717/peerj.21774/supp-11 Supplemental Information 11 RNAseq Expression Pattern *PtbHLH*.

## Additional Information and Declarations

### Data Availability

The following information was supplied regarding data availability:

The raw data are available in the Supplemental Files.

The data is available at Figshare:

- wang, xiaorui (2026). Transcriptome data of *Pinellia ternata* under MeJA, ABA and SA treatments. figshare. Dataset. https://doi.org/10.6084/m9.figshare.31697926.v1.

The raw sequence data are available at NGDC BioProject: PRJCA016404.
